# An optimised method for the proteomic profiling of full thickness human skin

**DOI:** 10.1186/s12575-016-0045-y

**Published:** 2016-07-21

**Authors:** Emily Bliss, Wendy E. Heywood, Malika Benatti, Neil J. Sebire, Kevin Mills

**Affiliations:** Centre for Translational Omics, UCL Institute of Child Health, 30 Guilford Street, London, WC1N 1EH UK; Histopathology Department, Great Ormond Street Hospital, London, WC1N 3JH UK

**Keywords:** Proteomics, Skin profiling, Mass spectrometry, Solubilisation

## Abstract

**Background:**

The skin is the largest organ of the human body and is the first line barrier defence against trauma, microbial infiltration and radiation. Skin diseases can be a result of multi-systemic disease or an isolated condition. Due to its proteolysis resistant properties there are relatively few human skin proteomic datasets published compared with other human organs or body fluids. Skin is a challenging tissue to analyse using traditional proteomic techniques due to its high lipid content, insolubility and extensive cross-linking of proteins. This can complicate the isolation and digestion of proteins for analysis using mass spectrometry techniques.

**Results:**

We have optimised a sample preparation procedure to improve solubilisation and mass spectral compatibility of full thickness skin samples. Using this technique, we were able to obtain data for the proteome profile of full thickness human skin using on-line two-dimensional liquid chromatography, followed by ultra-high definition label-free mass spectrometry analysis (UDMS^E^). We were able to identify in excess of 2000 proteins from a full thickness skin sample.

**Conclusions:**

The adoption of on-line fractionation and optimised acquisition protocols utilising ion mobility separation (IMS) technology has significantly increased the scope for protein identifications ten-fold.

**Electronic supplementary material:**

The online version of this article (doi:10.1186/s12575-016-0045-y) contains supplementary material, which is available to authorized users.

## Background

The skin, being the largest organ of our bodies is of paramount importance for barrier protection and first line defence against trauma, microbial infiltration and radiation. However when this barrier is damaged or diseased numerous dermatological conditions can arise. These diseases by their nature are superficial, visible and can look unpleasant, which can contribute to a significant psychological and social burden [1].

To understand more about diseases affecting the skin and the skin barrier in particular, it is important that we understand the complex composition of proteins, metabolites and lipids that make up the skin and how they play a functional role in its overall structure. The skin consists of three major distinct layers; the epidermis, dermis and the subcutaneous fat layer. The purpose of the subcutaneous fat layer is largely for insulation, the dermis is predominantly made up of collagens, elastin and fibrillin as well as nerve endings, sweat glands, sebaceous glands, hair follicles and major blood vessels providing nutrients for the epidermal layer [2]. The epidermis is the most superficial layer of the skin and comprises the skin barrier; despite being the thinnest of all three layers it is arguably the most complex [3].

Despite the importance of the skin there are relatively few human skin proteomic studies and datasets currently available in the literature [4] compared with other human organs or body fluids. A human-specific PubMed search shows that there are three times as many publications for “liver AND proteomics” [5] and twelve times as many publications for “blood AND proteomics” [6] compared with publications for “skin AND proteomics” [7]. Skin is a challenging tissue to analyse using traditional proteomic techniques due to its high lipid content, insolubility and extensive cross-linking of proteins. This can complicate the isolation and digestion of proteins for analysis using mass spectrometry techniques. Techniques used by research groups in the current selection of available publications about skin proteomics [7] include gel-based protein fractionation [8, 9], heavy isotope labelled assays to identify phosphorylated proteins [10] and studying the proteins secreted by the skin, rather than the skin tissue itself [11]. The latter two techniques are specific to a particular aspect of the skin proteome and do not represent the composition of the skin tissue as a whole. Gel-based protein isolation or fractionation techniques is a viable method for preparing samples prior to mass spectrometry analysis and one which we explored whilst developing the method presented in this paper. When comparing a gel-based fractionation technique and the on-line fractionation technique described here we found a 50 % increase in the number of proteins detected (data not shown).

Here we describe an optimised method of fractionating, chromatographically separating and detecting proteins from a trypsin digested sample of human skin. This method is relatively quick, consists of less sample preparation steps and less protein (3 μg) compared with similar gel-based methods.

## Results

**Fig. 1:**
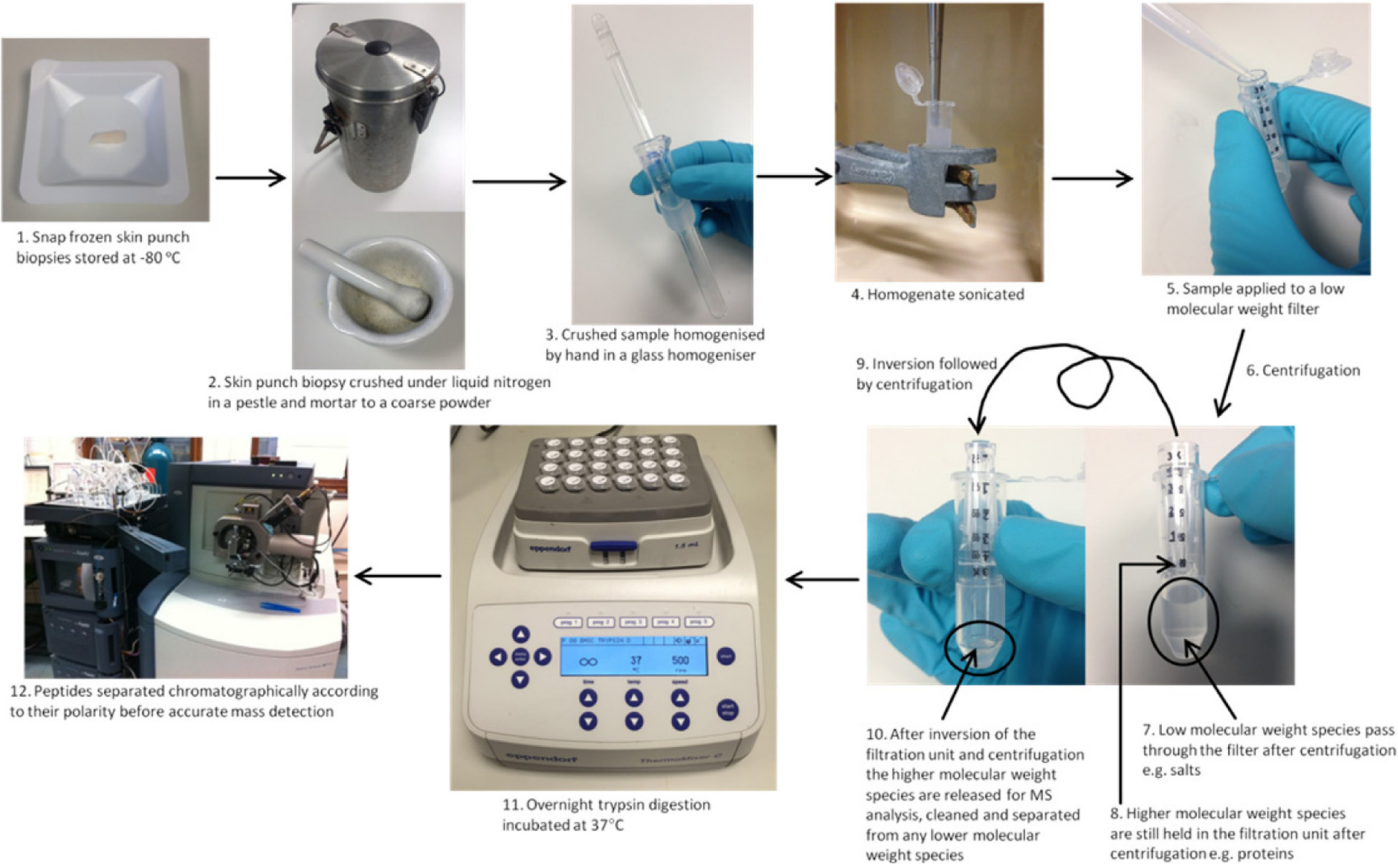
Diagram showing the generic workflow for preparing skin punch biopsies for MS analysis, including a low molecular weight species clean-up step. Points 1–12 show the sample preparation procedure that was originally used

Previously in our laboratory we have been able to identify 100–200 proteins from a full thickness skin biopsy [12], however with an upgraded system and modified methods we have improved this protocol and achieved a ten-fold increase in the number of identified proteins. In order to be a fair comparison the results presented in this section are from the same sample of skin tissue and are representative of the number of proteins detected by these methods from other samples of skin (data not shown). In the first instance the skin preparation method comprised of powdering the frozen tissue using a pestle and mortar, further homogenisation using a glass homogeniser, sonication, filtration of interfering low molecular weight compounds. This was a method adapted from other published proteomics methods for other tissues [13–15]. Peptides would then be separated using reverse phase chromatography a nanoAcquity liquid chromatography system coupled to a quadrupole time-of-flight mass spectrometer (QToF Premier, Waters, Manchester) [12] (Fig. 1). Using this workflow 218 proteins were identified from the skin samples as detailed in Additional file 1: Table S1.

**Fig. 2:**
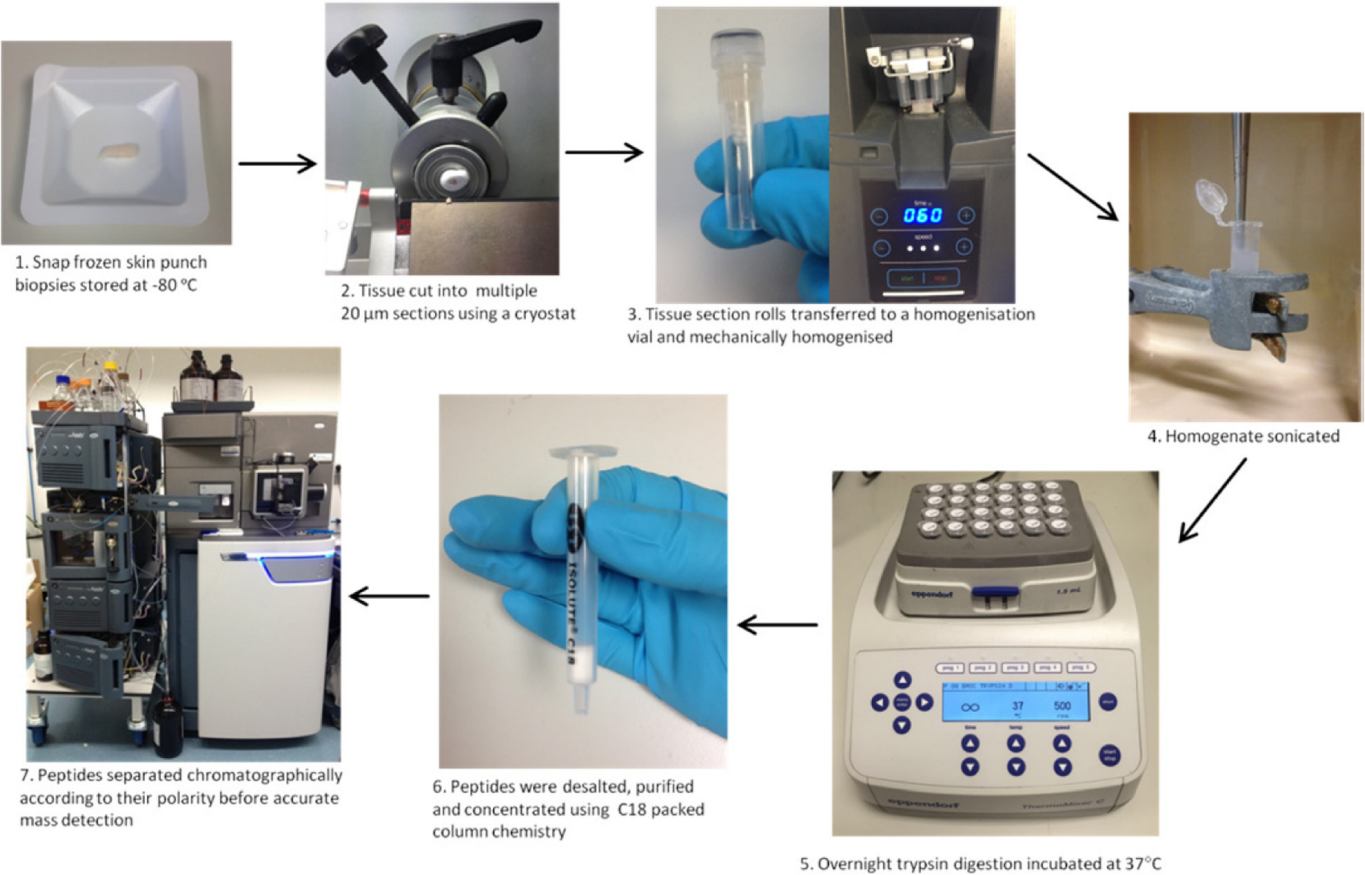
Diagram showing the modified workflow for preparing skin punch biopsies for MS analysis. Points 1–7 show the modified sample preparation procedure, which reduced sample preparation time and increased the depth of proteome coverage of the skin samples, compared with the previous workflow (Fig. 1)

Modifying the initial preparation method (Fig. 2) improved the number of detected proteins, to 2237 proteins, from the same sample of skin, those proteins are detailed in Additional file 2: Table S2.

**Fig. 3:**
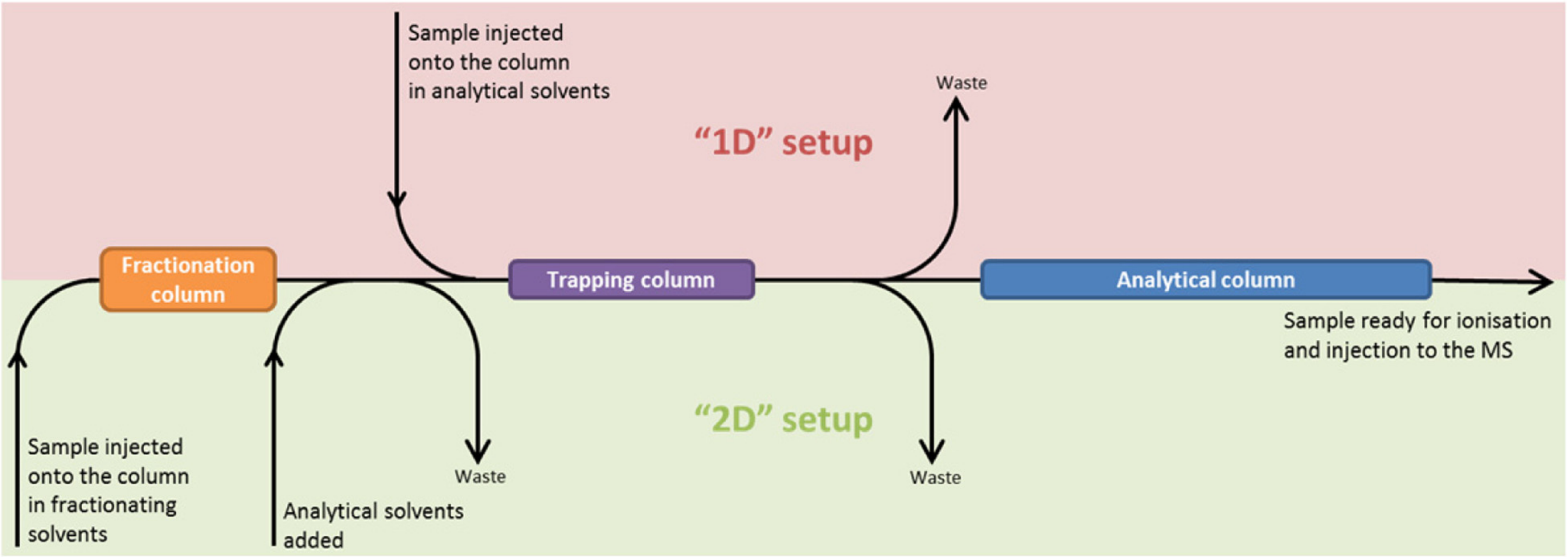
Figure detailing the original “1D” setup of the nanoAcquity and the upgraded “2D” setup including an additional module and column and the capability of on-line fractionating. This figure shows how in the original nanoAcquity LC “1D” setup the sample would be injected into the sampling loop, then be desalted on the trapping column, before being chromatographically separated on the analytical column. However in the upgraded “2D” setup there is an additional column after the sampling loop from which the sample is eluted from in individual fractions

Improved chromatographic separation was achieved using on-line fractionation technology [16] integrated with a nanoAcquity ultra-high performance liquid chromatography (UPLC) system (Fig. 3) rather that the shotgun approach of the initial method (Fig. 1).

One-dimensional (1D) separation is when the sample is injected onto the trapping column then the analytical column, the peptides are chromatographically separated according to polarity by an increasing concentration of acetonitrile over a 1 h gradient. Two-dimensional (2D) separation reduces the complexity of the sample by dividing it into multiple fractions, in this case eight on an additional fractionation column. Those fractions were then individually applied to the trapping column, then analytical column for chromatographic separation for the same 1 h acetonitrile gradient as the 1D samples and this was repeated for each individual fraction, totalling 8 hs of chromatographic separation for the 2D samples.

Further improvement in peptide quantitation and identification is achieved an optimised acquisition on a quadrupole time-of-flight mass spectrometer to include an additional ion mobility dimension of separation (SYNAPT G2-Si, Waters, Manchester) and capabilities of tailoring the collision energy ramp to the characteristic signature of peptides from this matrix (UDMS^E^).

## Discussion

In the first instance in our laboratory we analysed full thickness skin samples using the standard laboratory protocol, as shown in Fig. 1. That method allowed us to identify 218 proteins (Additional file 2: Table S1). We believed that this method could be altered to increase coverage of the skin proteome (Figs. 2 and 3). Two noticeably inefficient steps were the powdering and homogenisation. This was time consuming, not reproducible and inefficient (data not shown). Instead we chose to slice the skin using a cryostat into 20 μm sections and apply mechanical homogenisation using a ceramic bead homogeniser. We found this method to be quicker, more reproducible and efficient (data not shown). Another modification is the removal of the filtration step with a low molecular weight cut off filter. Instead to remove the majority of salts and hydrophobic compound such as lipids we used reverse phase carbon-18 (C18) pre-packed cartridges. C18 cartridges were selected because this is the same chemistry used in the solid phase of the on-line chromatographic separation thereby the samples are purified appropriately for the system.

Finally the liquid chromatography system was modified to include an additional binary solvent manager which allows the samples to be fractionated on-line using high pH/low pH fractionation (Fig. 3) [17, 18]. One-dimensional separation is quicker as it involved 1 h or chromatographic separation, whereas 2D separation reduced the complexity of the sample into eight separate fractions for analysis, totalling 8 hs of chromatographic separation and greater proteome coverage. The application of IMS to the mass spectral analysis also greatly improved this method. IMS in this case uses travelling-waves of helium gas against the flow of ions in order to separate co-eluting compounds based on shape and size giving each ion an unique ‘drift time’ measure thereby introducing an additional degree of separation of the ions [19, 20].

Having introduced these changes we have been able to identify 2237 proteins from a single skin sample (Additional file 2: Table S2), ten-fold greater than previously achieved in our group (Additional file 1: Table S1).

This method could equally be applied to other challenging or insoluble tissues such as formalin-fixed paraffin-embedded archived samples. Having developed this technique we will now be able to use the method to further study the skin in health and disease. We hope to be able to shed light on disease mechanisms and underlying biochemical changes that increase susceptibility to certain skin diseases. Further functional characterisation could also be achieved by physical separation of the skin layers. This is currently only achievable using laser capture technology that uses a very fine laser beam to cut out and collect sections of tissue from a microscope slide.

## Conclusions

In conclusion by modifying the sample preparation method, using a more complex liquid chromatography setup (2D-fractionation) and more recent mass spectrometry separation techniques (IMS) we have successfully been able to modify our original protocol in order to increase ten-fold the proteome coverage for skin samples.

## Methods

### Experiment design

We aimed to create a mass spectrometry-based method and workflow that would allow us to use proteomics to profile full thickness human skin. Using skin from surgically removed accessory digits, nanoflow UPLC online fractionation and separation before label-free UDMS^E^ raw data acquisition we were able to profile over 2000 proteins.

### Materials

All reagents and materials were purchased from Sigma-Aldrich, Poole, UK, unless stated otherwise. All solvents were of UPLC or higher grade specification.

### Human skin sample collection

The skin sample was obtained from the surface of an accessory digit surgically removed at Great Ormond Street Hospital, London. The skin was separated from the underlying tissue using a clean scalpel blade.

### Solubilisation of tissue and determination of protein concentration of the sample

Skin tissue was washed with a 1x phosphate buffer solution before being snap frozen in liquid nitrogen, embedded in optimum cutting temperature (Cell Path, Fisher Scientific), cryosectioned into 10 μm rolled tissue sections (Leica CM1860) and collected in a clean tube. The curls were suspended in 500 μL of 50 mmol/L ammonium bicarbonate containing 2 % w/v amidosulphobetanine-14. Curls and solution were transferred to a homogenisation vial (Precellys 0.5 mL tube containing 1.4 mm diameter ceramic beads, peqlab, VWR) and mechanically homogenised for 20 s at high power (Minilys®, Bertin Technologies). The solution was then transferred to ice for 1 min to stop thermal degradation. This was repeated a further two times. The vial was then incubated on ice for 1 h before a further three cycles of homogenisation. The homogenate was transferred to a clean tube and sonicated for 10 s using a Soniprep 150 sonicator (MSE UK). The sample was spun at 16,000 g for 10 min and the supernatant removed to a clean tube. A modified Lowry (Pierce™, ThermoFisher Scientific) protein assay was used to calculate the protein content of the sample.

### In-solution digestion of the homogenised sample

The equivalent of 50 μg of protein solution was taken and lyophilised using a freeze-drier. The pellet was reconstituted in 20 μL of 100 mmol/L trisma base, pH 7.8, containing 2 % w/v amidosulphobetanine-14, 6 mol/L urea and 2 mol/L thoiurea. To this solution 1.5 μL of a 1.94 mol/L dithioerythritol solution made up in 100 mmol/L trisma base, pH 7.8 was added, the sample vortexed, centrifuged briefly and incubated on a platform shaker at room temperature for 1 h. Three microlitres of a 1.94 mol/L2-iodoacetamide solution made up in 100 mol/L trisma base, pH 7.8 was added, the sample vortexed, centrifuged briefly and incubated at room temperature on a platform shaker for 45 min. The reaction volume was made up to 190 μL with Milli-Q water (Merck Millipore, Merck KGaA, Germany). Ten microlitres of 0.1 mg/mL Sequencing Grade Modified Trypsin (Promega, Madison, USA) was added, the sample vortexed briefly and incubated overnight at 37 °C.

### Purification of the digested sample peptides reverse phase C-18 chromatography

In order to remove the salt and lipid content of the sample, 100 μL of the digest solution was taken for a clean-up step using ISOLUTE® C18 columns (Biotage) was used.

### Two dimensional high pH fractionation of sample peptides, followed by low pH chromatographic separation using an online nanoAcquity ultra high performance liquid chromatography system

The lypholised sample was reconstituted in 25 μL of 3 % v/v acetonitrile containing 0.1 % v/v trifluoroacetic acid and 50 pmol/μL enolase peptides standard (MassPREP™, Waters) solution, centrifuged at 16,000 g for 10 mins and the supernatant transferred to a vial (TruView™ LCMS Certified, Total Recovery Vial, Waters). The nanoACQUITY UPLC (Waters, Manchester) system was configured in 2D with dilution set-up to allow for the first dimension to online fractionate the sample using high pH mobile phases directly before individual fractions entered the second dimension of chromatographic separation using low pH mobile phases. The first dimension was performed at 2 μL/min flow on an XBridge Peptide ethylene bridged hybrid C18 NanoEase Column (130 Å, 5 μm, 300 μm X 50 mm, 1/pkg (PN: 186003682), Waters, Manchester) mobile phase A was a 20 mmol/L ammonium formate, pH 9 solution and mobile phase B was 100 % acetonitrile. At the beginning of the first dimension 2.4 μL of the sample solution was loaded onto the XBridge Peptide column in 3 % mobile phase B for 1 min before a 4 min gradient up to 8.7 %, 11.8 %, 13.6 %, 15.3 %, 17.1 %, 19.3 %, 22.5 % and 50 % mobile phase B respectively for the 8 sequential fractions, after which the XBridge Peptide column was re-equilibrated at 3 % mobile phase B. After a fraction has been eluted from the XBridge Peptide column it enters the second dimension constituting low pH reverse phase chromatographic separation at 400 nL/min flow on an ACQUITY UPLC Peptide ethylene bridged hybrid C18 nanoACQUITY Column (10 Kpsi, 130 Å, 1.7 μm, 75 μm X 150 mm (PN: 186003543), Waters, Manchester) maintained at 35 °C, mobile phase A was 0.1 % v/v formic acid with 5 % v/v dimethyl sulphoxide and mobile phase B was 0.1 % v/v formic acid in 100 % acetonitrile with 5 % v/v dimethyl sulphoxide. Before the peptides from the first dimension are chromatographically separated they were diluted 1:10 during trapping with mobile phase A from the second dimension (20 μL/min 0.1 % formic acid with 5 % v/v dimethyl sulphoxide), concentrated and further desalted onto a nanoACQUITY UPLC Symmetry C18 Trap Column (100 Å, 5 μm, 180 μm x 20 mm, 2G, V/M (PN:186006527), Waters, Manchester). The second dimension gradient starts 20.5 min after the start of the first dimension at 3 % mobile phase B and increases to 40 % over 40 min, a further increase to 85 % mobile phase B occurs over the next 2 min and is held there for 2 min further, before returning to the starting conditions for 15 min of re-equilibration.

### Label-free UDMS^E^ mass spectrometry data acquisition

For each fraction a 60 min mass spectrometry analysis was performed on a SYNAPT G2-Si (Waters, Manchester) mass spectrometer in a UDMS^E^ mode in positive ion electrospray ionisation mode and operated in V-mode [21]. One second alternating high and low energy scans were performed at a capillary voltage of 3.0 kV, sampling cone voltage of 40 V, a source temperature of 70 °C over a mass range of 50–2000 Da in resolution analyser mode. Prior to fragmentation ion mobility separation was performed at a wave velocity of 650 m/s and a wave height of 40 V in order to separate similar precursor ions. The low energy scans were performed at a collision energy of 0 V and the high energy scans were performed on a gradient designed to use an optimised collision energy depending on the ion mobility bin, from 0–20 ion mobility bins the collision energy was 13.6 V increasing linearly to 49.1 V at 120 mobility bins, followed by another linear gradient to 54.1 V at 200 mobility bins. At a frequency of every 60 s lock mass of [glutamic acid^1^]-fibrinopeptide B is delivered via an auxiliary pump at 300 nL/min.

### Analysis and quantification of raw mass spectrometry files

Raw data were imported into Waters ProteinLynx GlobalServer version 3.0.1 in order to identify peptide masses corresponding to the fragmentation ion data. Mass corrections was applied based on the [glutamic acid^1^]-fibrinopeptide B mass delivered via an auxiliary pump. The processed spectra were merged prior to searching the UniProt reviewed human proteome. Database searching parameters were set to two fragment ions matched per peptide, four fragment ions per protein and two peptides per protein and one missed cleavage, fixed modifications were set to carbamidomethylation of cysteines and dynamic modifications of hydroxylation of aspartic acid, lysine, asparagine and proline and oxidation of methionine and the false discovery rate set to 4 %.

## Abbreviations

C18, carbon-18; IMS, ion mobility separation; QToF, quadrupole time of flight; UDMS^E^, ultra-high definition label-free mass spectrometry; UPLC ultra-high performance liquid chromatography

## Additional files

Additional file 1: Table detailing the proteins identifed in the original method (Fig. 1). (XLSX 19 kb) Additional file 2: Table detailing the proteins identified in the final method (Fig. 2). (XLSX 120 kb)
